# HHLA2 deficiency inhibits pancreatic cancer progression and THP-1 macrophage M2 polarization via EGFR/MAPK/ERK and mTOR/AKT pathway

**DOI:** 10.1186/s12957-024-03409-2

**Published:** 2024-05-18

**Authors:** Siqi Zhou, Zhangding Wang, Dian Zhao, Yao Fu, Shu Zhang, Zhiping Wang, Xiaoping Zou

**Affiliations:** 1 Department of Gastroenterology, Nanjing Drum Tower Hospital Clinical College of Jiangsu University, No.321, Zhongshan Road, Nanjing, 210008 China; 2 Department of Gastroenterology, Nanjing Drum Tower Hospital, The Affiliated Hospital of Nanjing University Medical School, Nanjing, 210008 China; 3 State Key Laboratory of Holistic Integrative Management of Gastrointestinal Cancers, National Clinical Research Center for Digestive Diseases, Xijing Hospital of Digestive Diseases, Fourth Military Medical University, Xi’an, 710032 China; 4 Department of Pathology, Nanjing Drum Tower Hospital, The Affiliated Hospital of Nanjing University Medical School, Nanjing, 210008 China; 5 Department of Anesthesiology, The Affiliated Hospital of Xuzhou Medical University, No.99, Huaihai West Road, Xuzhou, 221000 China

**Keywords:** Pancreatic cancer, HHLA2, Tumor-associated macrophages, Migration, Invasion

## Abstract

**Background:**

Human endogenous retrovirus subfamily H long terminal repeat associating protein 2, (HHLA2), a member of B7 family, exhibits heightened expression in various malignant tumors. However, the exact functions of HHLA2 in pancreatic cancer (PC) remain incompletely elucidated.

**Methods:**

We initially conducted an analysis of the B7 family members’ expression pattern in pancreatic tumor samples and adjacent normal tissues using The Cancer Genome Atlas (TCGA) database. Subsequently, immunohistochemistry, RT-qPCR and western blot methods were used to assess HHLA2 expression levels in PC tissues and cell lines. Furthermore, after silencing HHLA2 in PC cell lines, cell migration and proliferation of PC cells were detected by wound healing and CCK-8 assays, and cell invasion of PC cells was detected by transwell assays. We also investigated the regulation of epithelial-mesenchymal transition (EMT) markers and levels of EGFR, MEK, ERK1/2, mTOR and AKT via western blot analysis. Finally, the correlation between HHLA2 expression and immune infiltration was further explored.

**Results:**

Silencing of HHLA2 resulted in the inhibition of PC cell proliferation, migration and invasion, potentially through the suppression of the EGFR/MAPK/ERK and mTOR/AKT signaling pathway. Additionally, silencing HHLA2 led to the inhibition of M2-type polarization of tumor associated macrophages (TAMs).

**Conclusion:**

The knockdown of HHLA2 was observed to inhibit the migration and invasion of PC cells through the regulation of the EMT process and EGFR/MAPK/ERK and mTOR/AKT pathway. Furthermore, silencing HHLA2 was found to modulate M2 polarization of TAMs. These finding suggest that HHLA2 could be a promising therapeutic target for Pancreatic cancer.

**Supplementary Information:**

The online version contains supplementary material available at 10.1186/s12957-024-03409-2.

## Introduction

Pancreatic cancer (PC) is one of the most aggressive malignant tumors and known as ‘king of cancer’ [1, 2]. The incidence of PC ranks the eighth among all malignant tumors in China, and it is the seventh cause of cancer related death worldwide. Pancreatic ductal adenocarcinoma is predicted to become the second cause of cancer-related death in the next 20 years. The pancreas is located at the posterior side of the upper abdomen, which is easy to be ignored. Therefore, the early diagnosis of PC is difficult, and more than 80% of the first-diagnosed patients were at the advanced stage. Only 10% of patients with PC are suitable for surgery, and the risk of postoperative metastasis is also high, and patients with recurrence of PC are often highly resistant to radio and chemotherapies. Due to the lack of effective treatments, the 5-year survival rate of PC is less than 9% [3]. Therefore, results of current studies suggested that by regulating the composition of extracellular matrix, reactivation of the antitumor environment in PC may improve the therapeutic efficacy of current treatment resistance and benefit the patients [4].

Immune checkpoint plays an important role in the immune escape of tumor cells [5, 6]. Therapeutic strategies that inhibit the immune checkpoint signaling have been widely used in the treatment of PC. At present, the commonly analyzed immune checkpoint proteins mainly include cytotoxic T lymphocyte-associated antigen-4 (CTLA-4), programmed death receptor-1 (PD-1) and its ligands (PD-L1) [7, 8]. However, the therapeutic efficacy of immune checkpoints for the treatment of PC remains unclear. In a phase II clinical trial, 27 patients with PC did not respond to the treatment of anti-CTLA-4 monoclonal antibody Ipilimumab [9]; in another phase I trial using anti-PD-L1 monoclonal antibody, 14 patients with PC showed disappointing responses to the monotherapy [10]. Therefore, exploring additional immune checkpoint markers and developing adequate immune classifications to improve the efficacy of current anti-PC therapies is critical.

The B7 family proteins belong to the immunoglobulin superfamily (IgSF). The molecular structure of B7 family proteins is similar to that of the immunoglobulins. Each motif domain of B7 family proteins is a tightly folded structure composed of 70 to 110 amino acids, including IgV region and IgC2 region [11–13]. It is expressed as a single chain on the surface of antigen presenting cells (APCs) and acts as a second signal of immune response by binding to CD28 through IgV region [14]. B7 family members include B7-1 (CD80), B7-2 (CD86), B7-H1/PD-L1(CD274), ICOS-L/B7-H2(CD275), B7-H3(CD276), B7-H4, B7-DC/PD-L2(CD273), BT3.1(CD277) and the new member B7H7, which is also known as human endogenous retrovirus subfamily H long terminal repeat associating protein 2(HHLA2) [15]. uwhich is longer than most B7 family members. It is speculated that the HHLA2 protein contains an N-terminal signal peptide, which is mainly localized at the cell membranes and intracellular regions of different cells [16–18]. HHLA2 can activate TCR signaling and decrease the proliferation of the CD4 and CD8 cells, thereby decreased the production of IFN-γ, TNF-α, IL-5, IL-10, IL-13, L-17 A and IL-22 and reduced the immune responses [19, 20]. Results of previous studies suggested that HHLA2 may serve as a new immune checkpoint molecule for the development tumor and participate in the process of tumor immune escape, and provide opportunities for the survival of tumor cell [21]. However, the underlying mechanism remains unclear.

At present, there are few reports focused on the roles and mechanisms of HHLA2 in PC. In this study, we detected the expression of HHLA2 in PC tissues, performed cell studies and also used bioinformatics to explore the roles of HHLA2 in PC. We hypothesized that HHLA2 is over-expressed in PC and may facilitate the development of the tumor.

## Materials and methods

### Public data information

We retrieved transcriptome RNA expression data and corresponding clinical information of 345 PC patients from TCGA (The Cancer Genome Atlas) (http://cancergenome.nih.gov/) database. Inclusion criteria: confirmed PC diagnosis, availability of complete gene expression profiles, and comprehensive prognostic follow-up data. We employed the limma and SVA packages to homogenize the transcriptome data from various genes, and the combined database was referred to as Merge database. Subsequently, E-MTAB-5639 consisting RNA-seq on subcutaneous PDTXs from 24 PDAC samples, was used to verify the differentially expressed genes (DEGs) between different HHLA2-expression groups. To assess immune cell infiltration in PC tumor tissues, TIMER (Tumor Immune Estimation Resource) (http://timer.cistrome.org/) database was utilized to determine the infiltration of immune cells in tumor tissues TIMER provide valuable information regarding the infiltration of B cells, CD4 + T cells, CD8 + T cells, neutrophils, macrophages and dendritic cells, offering insights into the immune landscape of PC.

### Human specimens

Sixty-two PC tissues and adjacent normal pancreas were collected at the affiliated Drum Tower Hospital of Nanjing University Medical School. Writing informed consent was acquired from the donors. The study was approved by the Institutional Ethics Review Board of the affiliated Drum Tower Hospital of Nanjing University Medical School.

### Cell lines and reagents

Human normal Pancreatic Duct Epithelial cell line (HPDE), human pancreatic cancer cell lines (BxPC-3 CFPAC-1, Capan-2, PANC-1, HPAC, AsPC-1, MIAPaCa-2 and SW1990) were purchased from American Type Culture Collection (American Type Culture Collection cell line bank, Manassas, VA, USA). DMEM medium and fetal bovine serum was purchased from Gibco (USA). HHLA2 siRNA and negative control siRNA (NC siRNA) plasmids were designed and synthesized by Genepharma (Shanghai, China). Transfection reagent Lipofectamine 2000 was purchased from Thermo Fisher (San Jose, CA, USA). DAB chromogen was purchased from Sangon (Shanghai, China).

### Cell culture

Cells were cultured in DMEM medium containing 10% FBS fetal bovine serum at 37℃ in a 5% CO_2_ environment. The cells were digested and passaged when reached about 80% confluency. For the cell passage, the cells were digested with trypsin and then collected by centrifugation. Cells were then resuspended in fresh medium, and the cell suspension was ready to seed plates as required for subsequent experiments.

### Survival analysis

The expression and clinical data of B7 family members including CD274, CD276, CD80, CD86, HHLA2, ICOSLG, NCR3LG1, PDCD1LG2, VSIR and VTVN1 in PC patients were analyzed. According to the expression levels of B7 family members, the first 30% and the last 30% were set as high expression group and the low expression group, respectively. Kaplan-Meier analysis was used to compare the survival rate of PC patients between high and low expression of B7 family members.

### Immunohistochemistry

Clinical specimens of PC were fixed in 10% formalin and encapsulated in paraffin blocks. 5-µm sections were cut and mounted on slides. Samples were blocked with 5% goat serum for 1 h at room temperature, followed by incubation with the primary antibodies (1:200) overnight at 4℃. After washing twice with PBS, rabbit secondary antibodies (1:200) were incubated for 2 h at room temperature. Then, 3, 3’ diaminobenzidine (DAB) was used for coloration, hematoxylin counterstained, dehydration and sealing. Sections incubated with goat serum without the primary antibody served as negative controls.

### Colony-formation assay

PC cells transfected with siRNA were counted and seeded into 6-well plates with 1000 cells/well. After 14 days, the cells were fixed in methanol and colonies were counted after staining with 0.1% crystal violet in 25% methanol for 20 min. The results were presented as the average number of counted colonies per well under each condition. All the experiments were performed in triplicate and repeated three times independently.

### Wound healing assay

Cells were seeded onto 6-well plates at a density of 5${\times}$10^5^ cells/well and cultured overnight in an incubator. After the cells were covered with the culture plate, the tip of a 20 µl pipette gun was used to scratch the cell monolayer perpendicular to the plate, with an interval of 0.5 ~ 1 cm. After washing the cells with PBS for 3 times, the medium containing 2% FBS and siHHLA2-2 and siHHLA2-3 were added, and then the cells were cultured in an incubator. Measurements were taken at the same location after 0 h and 24 h of culture, and three compound wells were measured each time and repeated three times. Scratch width was measured using Image J. Wound healing rate= (0 h scratch width- 24 h scratch width)/ 0 h scratch width $\times 100{\%}.$  

### Transwell assay

Cellular motility and invasive abilities were determined using Transwell (8.0 μm polycarbonate Membrane, Corning Life Sciences, Bedford, MA, USA) and Matrigel invasion (BD Biosciences, San Jose, CA, USA), respectively. For the cell invasion analysis, transwell chambers were placed on 24-well plates; the upper chamber coated with Matrigel was seeded with 2 × 10^5^ cells and cultured with 200 µl serum-free medium, and the lower chamber was added with 600 µl DMEM medium containing 10% FBS. After 24 h of culture, the chambers were removed and fixed with 4% paraformaldehyde at room temperature for 20 min, and then stained with crystal violet at room temperature for 15 min. The cells in the upper chamber were gently wiped off with a cotton swab. Five fields were randomly selected under a microscope and photographed (200$\times$), Image J software was used for cell counting. The migration experiments were performed in the same manner, but the upper chamber was without the Matrigel.

### Western blot

Total protein was extracted from cell samples, and protein concentration was determined by BCA assay. Proteins were then denatured by boiling with 20 µg protein loading buffer and wet transferred to nitrocellulose membrane after gel electrophoresis on 10%SDS-PAGE, blocked with 5% skim milk powder for 1 h at room temperature. After addition of primary antibodies (1:1000), the membranes were incubated overnight at 4 ℃, washed, and the secondary antibodies (1:2000) were added and incubated for 2 h at room temperature., Then the membranes were washed, and chromogen was added. Protein bands were visualized using a chemiluminescence imaging system.

### Functional enrichment and Immuno-infiltration analysis

The GO (Gene Ontology) enrichment analysis was performed to determine significantly enriched GO terms for the differentially expressed genes. In order to investigate any changes in biological functions and related pathways, HALLMARK gene set (“H.all.v7.4.symbols.gmt” was downloaded from the Molecular Signatures Database as a reference gene set, http://www.gsea-msigdb.org/gsea/downloads.jsp), and KEGG (Kyoto Encyclopedia of Genes and Genomes) pathway enrichment analysis were performed. Enrichment analysis was performed by the R package clusterProfiler (version 3.14.3). P value of < 0.05 was considered statistically significant. Sangerbox Tools [22] was used to calculate the relationship between each B7 family members and significantly infiltrated immune cells. In addition, TCGA and TIMER2.0 databases were used to analyze the correlation between M2 macrophage infiltration and HHLA2 expression in PC.

### Immune correlation analysis of HHLA2

Spearman coefficient was used for evaluating the correlation between HHLA2 and neutrophil and macrophages M2. At the same time, the results were visualized in the form of scatter plots. *P* value < 0.05 was considered as a statistically significant threshold.

### THP-1 differentiation and coculture system

To be differentiated into adherent macrophages, THP-1 cells (5 × 10^5^/well) were seeded into 6-well plates in the presence of 100 ng/ml of phorbol 12-myristate 13-acetate (PMA, Sigma) for 24 h. The differentiated macrophages were named as M0 macrophages. To establish a co-culture system, we transfected AsPC-1 and Capan-2 cells with siHHLA2 or NC. At 48 h post-transfection, we used the culture supernatant to incubate the M0 macrophages. After 48 h of co-culture, the macrophages were harvested for analysis.

### Statistics analysis

Data were analyzed using SPSS software and GraphPad Prism 7 software. Data are presented as mean ± standard deviation (SD) or 95% confidence intervals. The continuous variables were compared between the two groups by unpaired t test, and the comparison between multiple groups was performed by one-way ANOVA followed by Tukey’s test. Categorical variables were compared with the use of the chi-square test or Fisher’s exact test, as appropriate. Correlations between two groups were analyzed by the Spearman rank-order correlation analysis. Kaplan-Meier method was used to draw survival curves. *p* < 0.05 was considered to indicate a statistical difference.

## Results

### Comparison of the expressions of B7 family members between PC tissues and normal tissues

**Fig. 1:**
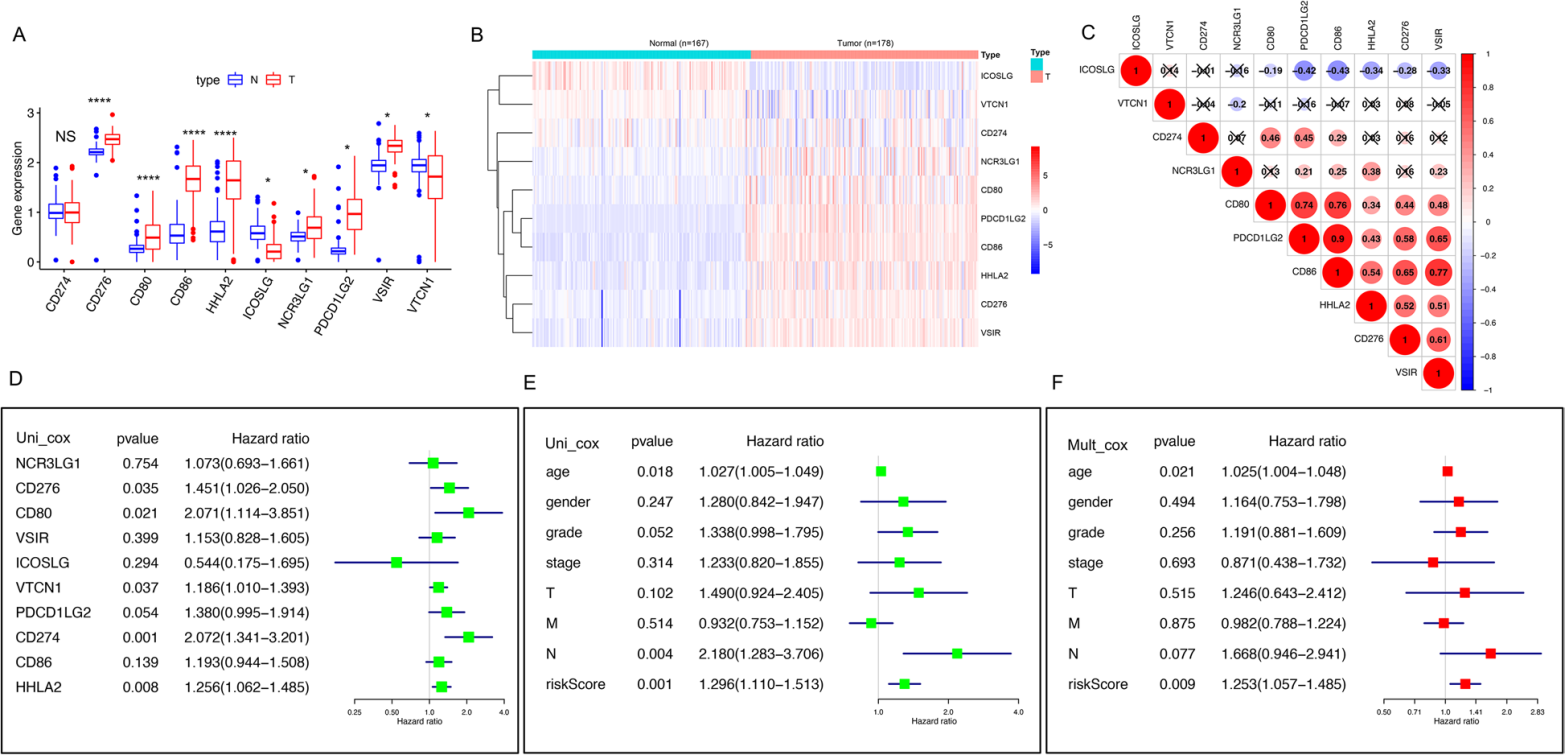
The expressions of B7 family members between PC tissues and normal tissues. **A** Expression of B7 family members in pancreatic cancer tissues and normal tissues. **B** Heatmap of B7 family member in normal and cancer tissues (green: low-expression; red: high-expression). **C** Spearman correlation analysis for the 10 types of B7 family members (red: positive correlation; blue: negative correlation). **D** Forest plot of B7 family members by univariate Cox regression analysis. **E**&**F** Univariate and multivariate Cox regression analysis of clinical parameters and risk score

First, we combined the TCGA and the GTEx datasets and analyzed the gene expression data of 178 tumors and 167 normal pancreatic tissues. The results were presented as boxplots (Fig. 1A) and heatmap (Fig. 1B). We found that CD276, CD80, CD86, HHLA2, NCR3LG1 and PDCD1LG2 were significantly elevated, while ICOSLG expression was significantly decreased in PC tissues. And the expression of CD274 has no significant differences between tumor tissues and normal tissues. Additionally, a correlation network plot was used to unravel the correlation features among these 10 B7 family members. Figure 1C showed the correlations between the above genes. Concomitantly, we studied the effects of these 10 B7 family members, age, gender, grade, stage, T/M/N classification and risk score on prognosis of pancreatic cancer. In univariate Cox regression analysis, CD276, CD80, ,VTVN1, CD274and HHLA2 were high-risk factors. The hazard ratio (95% CI) of CD276, CD80, CD274, HHLA2 was 1.451 (1.026,2.050), 2.071 (1.114, 3.851), 1.186 (1.010,1.393), 2.072 (1.341,3.201) and 1.256 (1.062, 1.485), respectively (Fig. 1D). In TCGA dataset, the univariate Cox regression analysis found that age, stage of N and riskScore were significantly associated with survival but the corresponding multivariate Cox regression analysis found that only age and riskScore were significantly associated with survival (HR = 1.296, 95% CI = 1.110–1.513, *p* = 0.001, and HR = 1.253, 95% CI = 1.057–1.485, *p* = 0.009, respectively; Fig. 1E&F).

### The expressions of B7 family members were correlated with the survival of PC patients

**Fig. 2:**
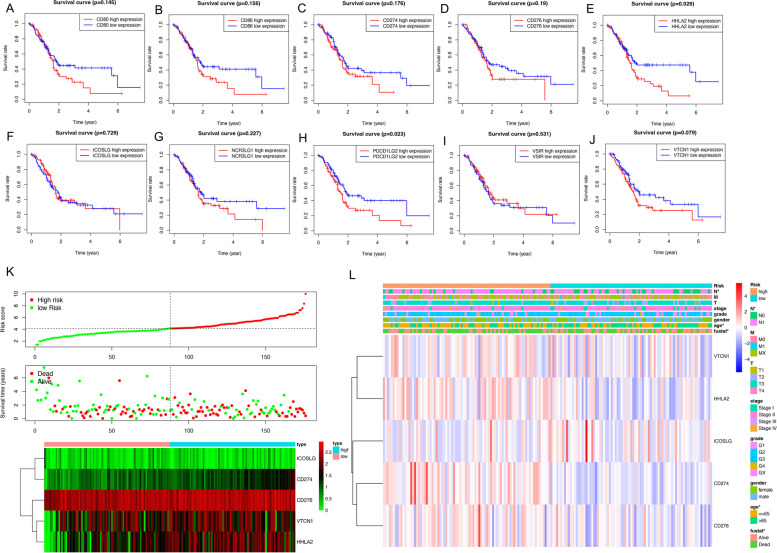
The B7 family members were correlated with the survival of PC patients. **A-J** Survival curves of B7 family members and prognosis of PC; **K** The distribution of risk scores, survival status and heatmap of the selected genes expression levels in datasets of TCGA-PC (left of the dotted line: low-risk population; right of the dotted line: high-risk population; distribution of patients based on the median risk score.) **L** Correlation heatmap of B7 family members and clinicopathological features in datasets of TCGA-PC

Next, the potential prognostic values the B7 family members were further evaluated by KM survival analysis based on TCGA datasets. The results showed that high expression of B7 family members in PC predicted poor survival rate. Moreover, increased expression of HHLA2 and PDCD1LG2 were significantly correlated with the unfavorable survival in PC (Fig. 2A-J). Furthermore, we calculated a risk score for each patient based on the gene expression level and the risk coefficient of each gene. The prognostic value of the risk score was assessed by comparing the difference in survival between the high-risk and low-risk groups. In the univariate regression analysis described earlier, we selected 4 high-risk genes CD274, CD276, VTVN1, and HHLA2 and 1 low-risk gene ICOSLG for risk scoring. We plotted the distribution of patients’ risk scores and overall survival status as well as the expression profiles of the five genes. As shown in the Fig. 2K, significantly higher mortality and a shorter OS time were observed in the high-risk group. The heatmap showed that the expression levels of VTVN1 and HHLA2 in the low-risk group were lower than high-risk group. Moreover, a heatmap was used to demonstrate the correlation between B7 family members and clinicopathological features in TCGA-PC: that patients in the high-risk group were older and had more lymph node metastases compared to the low-risk group (Fig. 2L).

### HHLA2 was over-expressed in PC

**Fig. 3:**
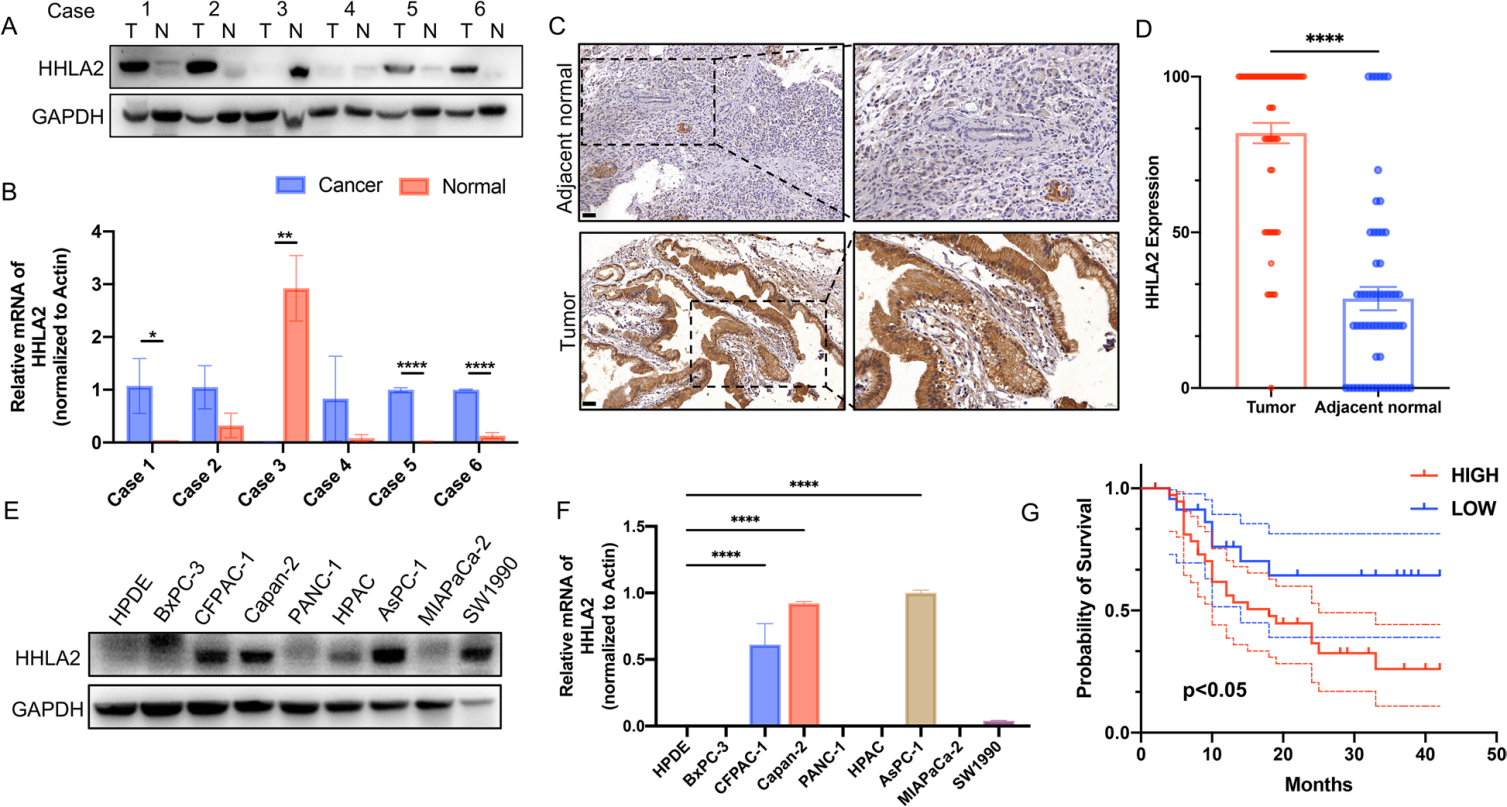
The expression level of HHLA2 in pancreatic cancer tissues and cells. **A** The expression of HHLA2 in pancreatic cancer tissue was detected by WB. **B** The expression of HHLA2 in pancreatic cancer tissue was detected by RT-qPCR. **C**&**D** The expression of HHLA2 in 62 paired adjacent normal tissues and tumor tissues was detected by IHC. Scale bar: 50 μm. **E**&**F** The expression of HHLA2 in pancreatic cancer cells was detected by WB and RT-qPCR. **G** Differential expression of HHLA2 in PC tissues and normal tissues and its survival curve with patients were analyzed. ^*^p<0.05, ^**^p<0.01, ^***^p<0.001, ^****^p<0.0001

Furthermore, the expression of HHLA2 in 6 paired PC tissues and the adjacent normal tissues were detected by WB and RT-qPCR. We found that the expression of HHLA2 in PC tissues was significantly higher than that in adjacent normal tissues (Fig. 3A&B, *p* < 0.01). The IHC staining of tissue samples from 62 PC patients further verified the HHLA2 protein expression (Fig. 3C). Statistical analysis showed that HHLA2 expression in tumor tissues was significantly higher than that in adjacent normal tissues (Fig. 3D). (*P*<0.05). In addition, Kaplan–Meier (KM) survival analysis showed that higher expression of HHLA2 was significantly associated with a poor survival outcome in 62 PC patients (Fig. 3G, *p* < 0.05).The protein and mRNA expression levels of HHLA2 were detected in normal ductal epithelial cell line of pancreas and different human pancreatic cancer cell lines by WB and RT-qPCR. Capan-2 and AsPC-1 cells with high expression of HHLA2 were selected for subsequent experiments (Fig. 3E&F, *p* < 0.01).

### Relationship between HHLA2 expression and clinic pathological features of PC patients

In the data of 171 PC patients obtained from TCGA database and our self-made paraffin chip (including 62 PC patients), the PC patients were divided into HHLA2 high and low expression groups. As shown in supplemental Table S1, in TCGA database, there was no significant correlation between HHLA2 expression and age, gender, or tumor TNM stage of the patients (*p* > 0.05). In our self-made paraffin chip, we can see that there was significant correlation between HHLA2 expression and overall survival status.

### Effects of siHHLA2 on viability and proliferation of PC cells

**Fig. 4:**
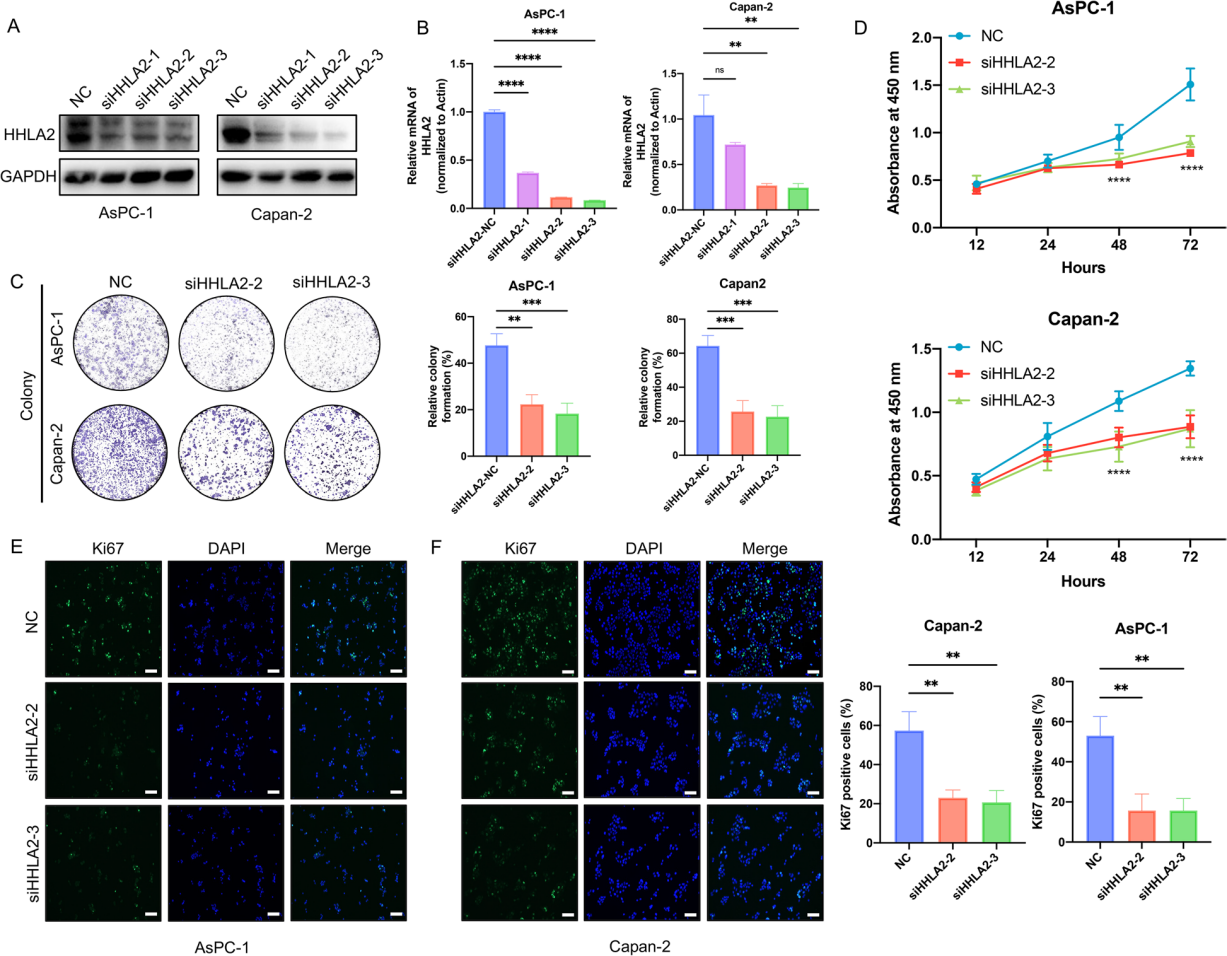
Effects of siHHLA2 on viability and proliferation of PC cells. **A **The relative expression level of protein of siHHLA2 in AsPC-1 and Capan-2. **B** The relative expression level of mRNA of siHHLA2 in AsPC-1 and Capan-2. The effect of siHHLA2-2 and siHHLA2-3 on the growth of PC cells was detected by colony formation assay (**C**), CCK-8 (**D**) and immunofluorescence staining. Scale bar: 100 μm (**E**&**F**). ^*^p<0.05, ^**^p<0.01, ^***^p<0.001, ^****^p<0.0001

Then, siHHLA2-1, siHHLA2-2 and siHHLA2-3 were transfected into AsPC-1 and Capan-2 cells, and the protein and mRNA expression of HHLA2 was detected by WB and RT-qPCR. Compared with the NC control group, siHHLA2-2 and siHHLA2-3 markedly inhibited the relative expression of HHLA2 in AsPC-1 and Capan-2 cells(*p* < 0.01) while siHHLA2-1 showed low inhibitory effects on HHLA2 expression in Capan-2 cells (Fig. 4A&B, *p* > 0.05). Therefore, siHHLA2-2 and siHHLA2-3 were used in the following studies. The colony formation assay showed that siHHLA2 inhibited the proliferation of AsPC-1 and Capan-2 cells (Fig. 4C, *p* < 0.001). Furthermore, results of CCK-8 suggested that compared with NC group, siHHLA2 significantly inhibited the viability of PC cells AsPC-1 and Capan-2 (Fig. 4D, *p* < 0.05). Furthermore, the immunofluorescence staining demonstrated that after siHHLA2 treatment, the expressions of Ki67 significantly decreased (Fig. 4E&F, *p* < 0.05). Taken together, these data indicate that knockdown of HHLA2 expression can inhibit the viability of human gastric cancer cells.

#### Function Enrichment Analysis of HHLA2 in PC

**Fig. 5:**
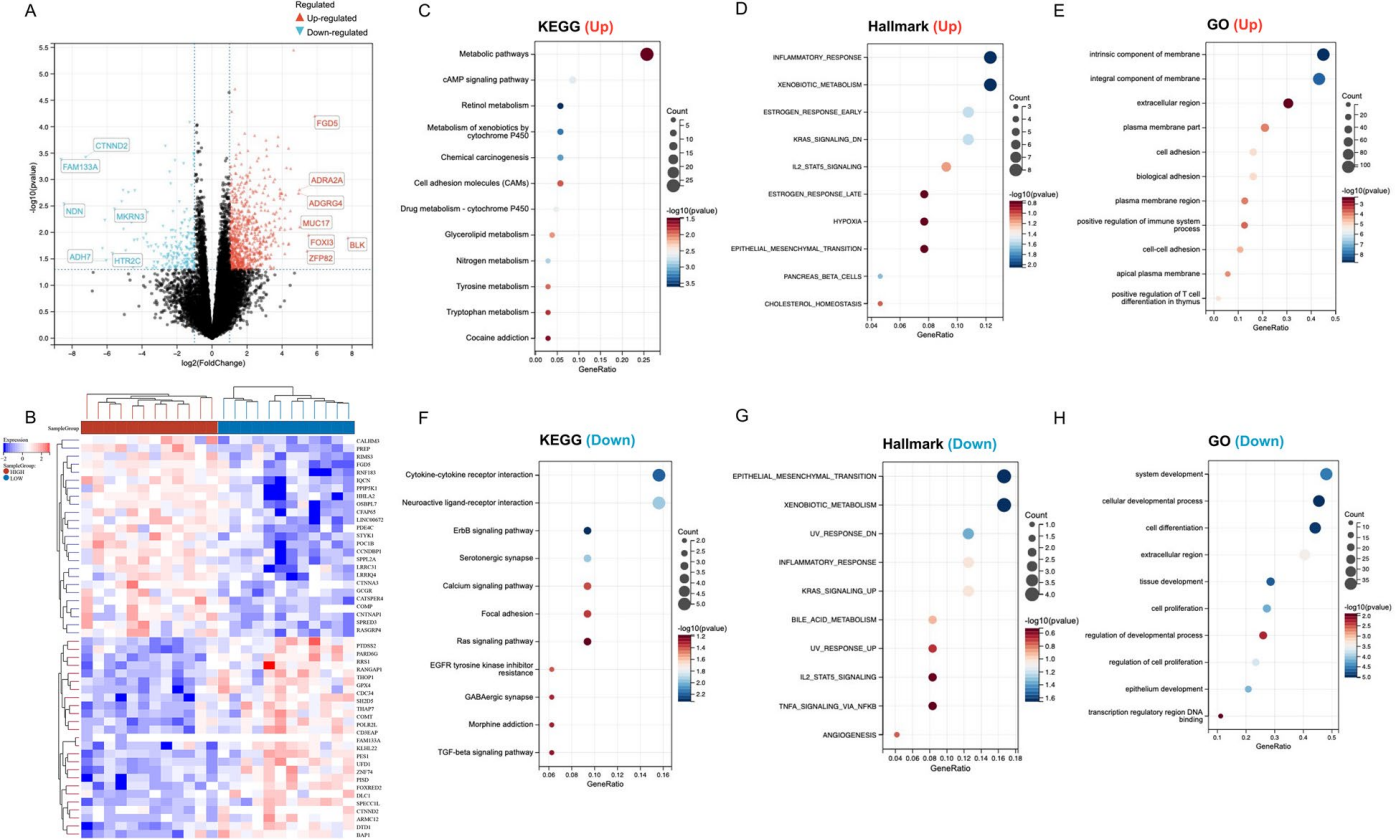
Function enrichment analysis of HHLA2-related gene sets in PC. **A** Differentially expressed genes (DEGs) for high expression of HHLA2 vs. low expression of HHLA2 in PC were shown in the volcano plot, with red dots representing significantly up-regulated genes and blue dots representing significantly down-regulated genes in PC with high expression of HHLA2. **B** The heatmap exhibits the expression level. **C** Enrichment analysis for KEGG pathway of up-regulated genes. **D** Enrichment analysis for Hallmark pathway of up-regulated genes. **E** Enrichment analysis for GO term of up-regulated genes. **F** Enrichment analysis for KEGG pathway of down-regulated genes. **G** Enrichment analysis for Hallmark pathway of down-regulated genes. **H** Enrichment analysis for GO term of down-regulated genes

Based on the above, we found that the expression level of HHLA2 was significantly correlated with the proliferative ability of pancreatic cancer cells. However, the signal pathway changes caused by HHLA2 in PC are unknown. In order to deepen the understanding of the functional meaning of HHLA2 in PC, the functional enrichment analysis for HHLA2-related gene sets in PC was performed. We first identified 918 differentially expressed genes (DEGs) between HHLA2-high and HHLA2-low expression groups using the E-MTAB-5639 cohort, of which the HHLA2 high-expression group had 724 up-regulated genes and 194 down-regulated gene expression groups compared to the HHLA2 low-expression group (Fig. 5A&B). KEGG analysis showed that up-regulated genes are involved in Cell adhesion molecules and metabolism-related pathways, including Retinol metabolism, Glycerolipid metabolism, Nitrogen metabolism, and Tyrosine metabolism (Fig. 5C). Hallmark analysis showed correlations with the Inflammatory response and Epithelial Mesenchymal Transition (EMT) pathways (Fig. 5D). GO term analysis showed that upregulated genes were significantly enriched in cell adhesion-related pathways (Fig. 5E). For down-regulated genes, KEGG analysis showed that these genes were involved in the Cytokine-cytokine receptor interaction and EGFR-related signaling pathways such as the ErbB signaling pathway and the Ras signaling pathway (Fig. 5F). Hallmark analysis showed correlation with EMT pathway and Inflammatory response (Fig. 5G), similar to up-regulation genes. GO term analysis also showed that down-regulated genes were enriched during cellular development process and closely related to cell proliferation and epithelium development (Fig. 5H). Above results implied that HHLA2 might be involved in the occurrence and development of PC through EMT pathway and EGFR-related signaling pathway.

### Effects of siHHLA2 on the migration and invasion of PC cells

**Fig. 6:**
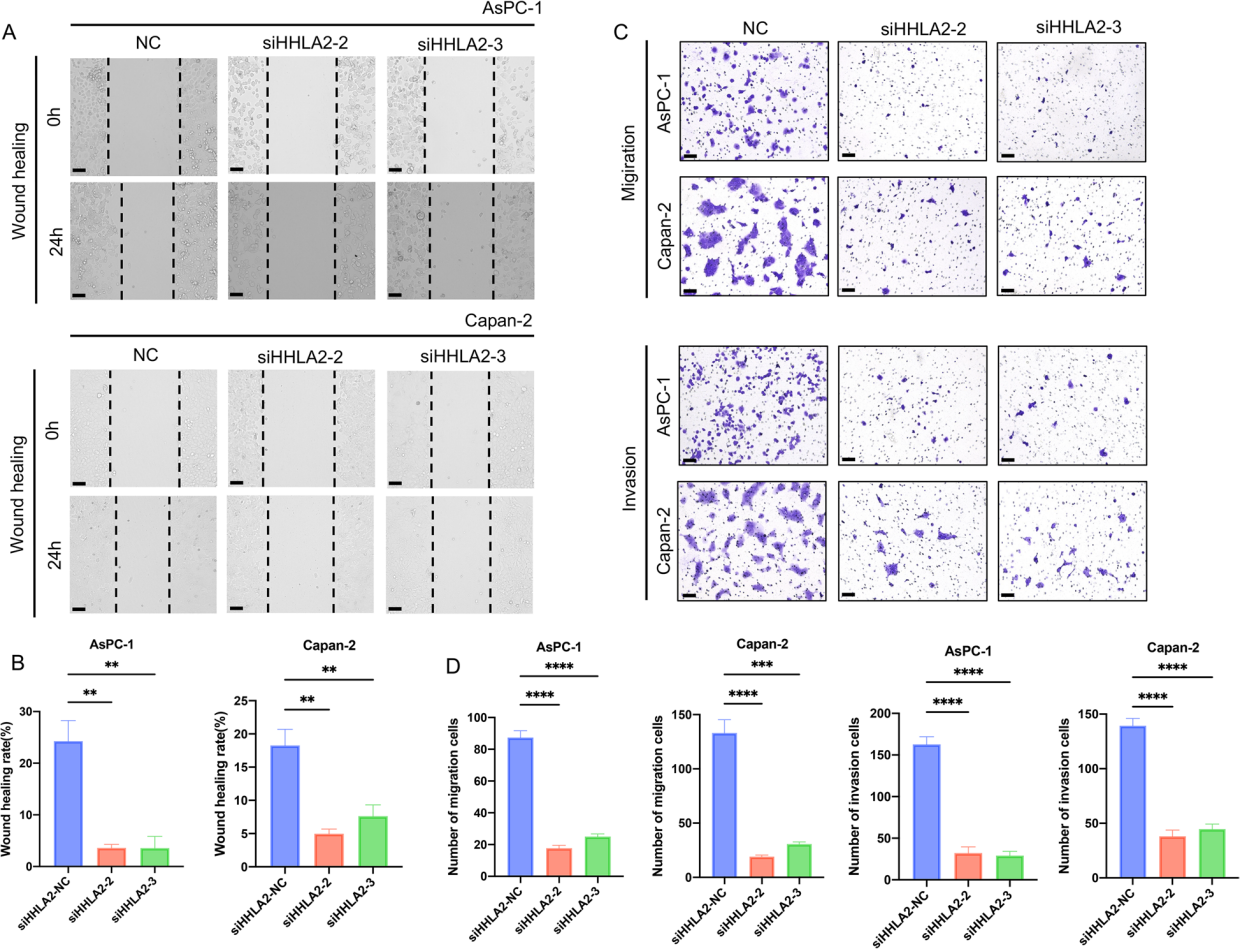
Effects of siHHLA2-2 and siHHLA2-3 on the invasion and migration. **A**&**B**, migration and invasion (**C**&**D**) of AsPC-1 and Capan-2 cells. Scale bar: 100 μm. ^*^p<0.05, ^**^p<0.01, ^***^p<0.001, ^****^p<0.0001

To clarify the effects of HHLA2 on PC cell metastasis, wound healing and transwell assays have been conducted. As shown in Fig. 6A and B, compared with the NC group, the wound healing rate of siHHLA2-2 group and siHHLA2-3 group in AsPC-1 and Capan-2 were significantly decreased after 24 h. Meanwhile, the results of transwell assays indicated that siHHLA2 significantly reduced the number of invaded PC cells (Fig. 6C&D).

### Effects of siHHLA2 on mTOR / AKT and EGFR/MAPK/ERK signaling pathway in PC cells

**Fig. 7:**
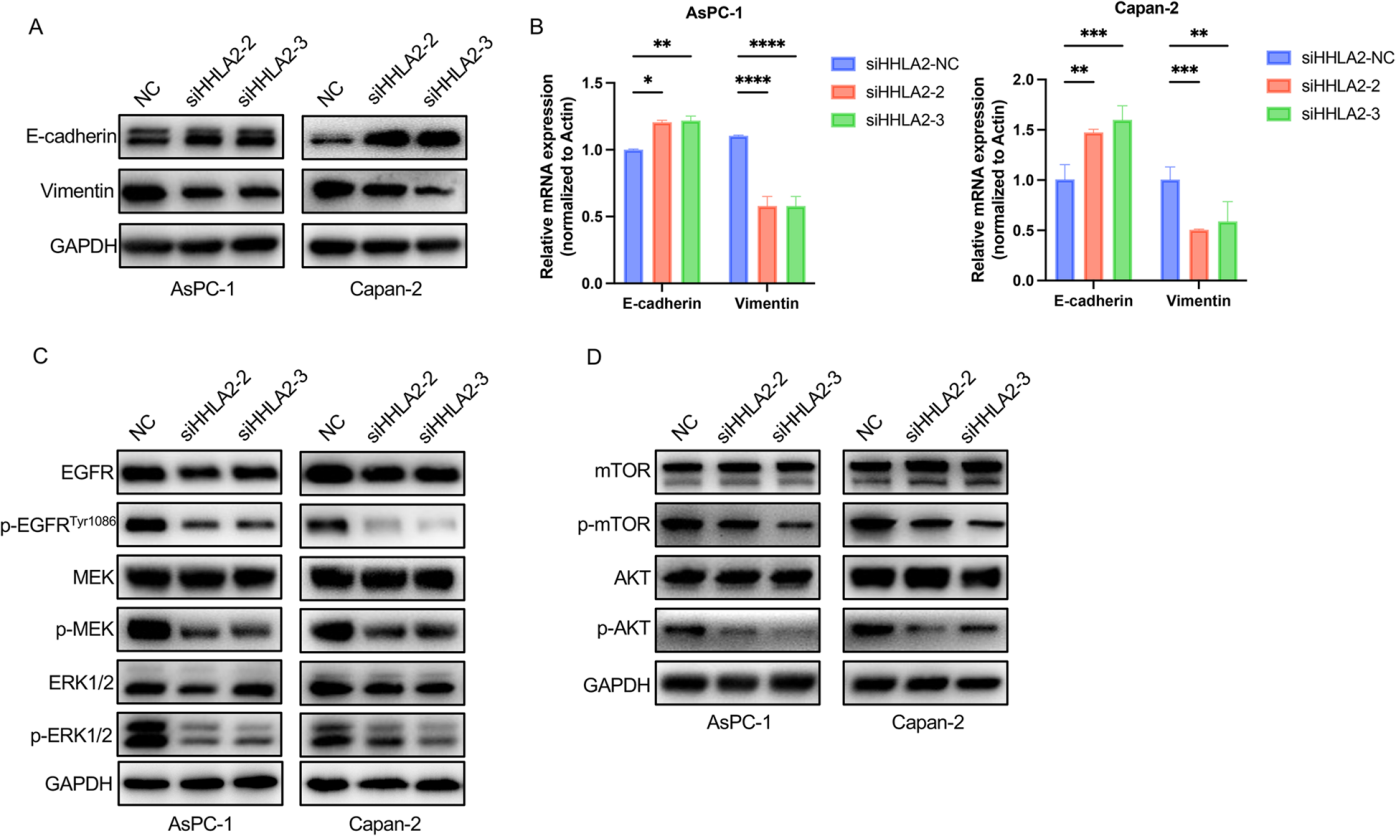
Effects of siHHLA2-2 and siHHLA2-3 on EGFR/MEK/ERK1/2/AKT/mTOR pathway in AsPC-1 and Capan-2 cells. **A**&**B** WB and RT-qPCRwere performed to evaluate the protein and mRNA levels of E-Cadherin and Vimentin expression after HHLA2 knockdown. **C** Knockdown of HHLA2 inhibited the activity of EGFR/MAPK/ERK signaling pathway in AsPC-1 and Capan-2 cells. **D** Knockdown of HHLA2 inhibited the activity of AKT/mTOR signaling pathway in AsPC-1 and Capan-2 cells. ^*^p<0.05, ^**^p<0.01, ^***^p<0.001, ^****^p<0.0001

Next, the effects of siHHLA2 on EMT of PC cells were examined. WB and RT-qPCR results showed that the expression of epithelial marker E-cadherin was increased, and the expression of mesenchymal marker vimentin was inhibited by the intervention of siHHLA2 (Fig. 7A&B). EGFR is a cell surface receptor that plays a vital role in regulating tumor metastasis by activating downstream signaling pathways, including the MAPK pathway and the PI3K/Akt pathway. Then, we examined the phosphorylation levels of ERK1/2, MEK, EGFR, AKT and mTOR. The expressions of p-EGFR, p-MEK, p-ERK1/2, p-AKT and p-mTOR were all significantly decreased compared with NC group (Fig. 7C&D). These results suggested that knockdown of HHLA2 inactivated EGFR/MAPK/ERK and mTOR/AKT pathway.

### Silencing of HHLA2 in PC cells inhibits M2 polarization of macrophages

**Fig. 8:**
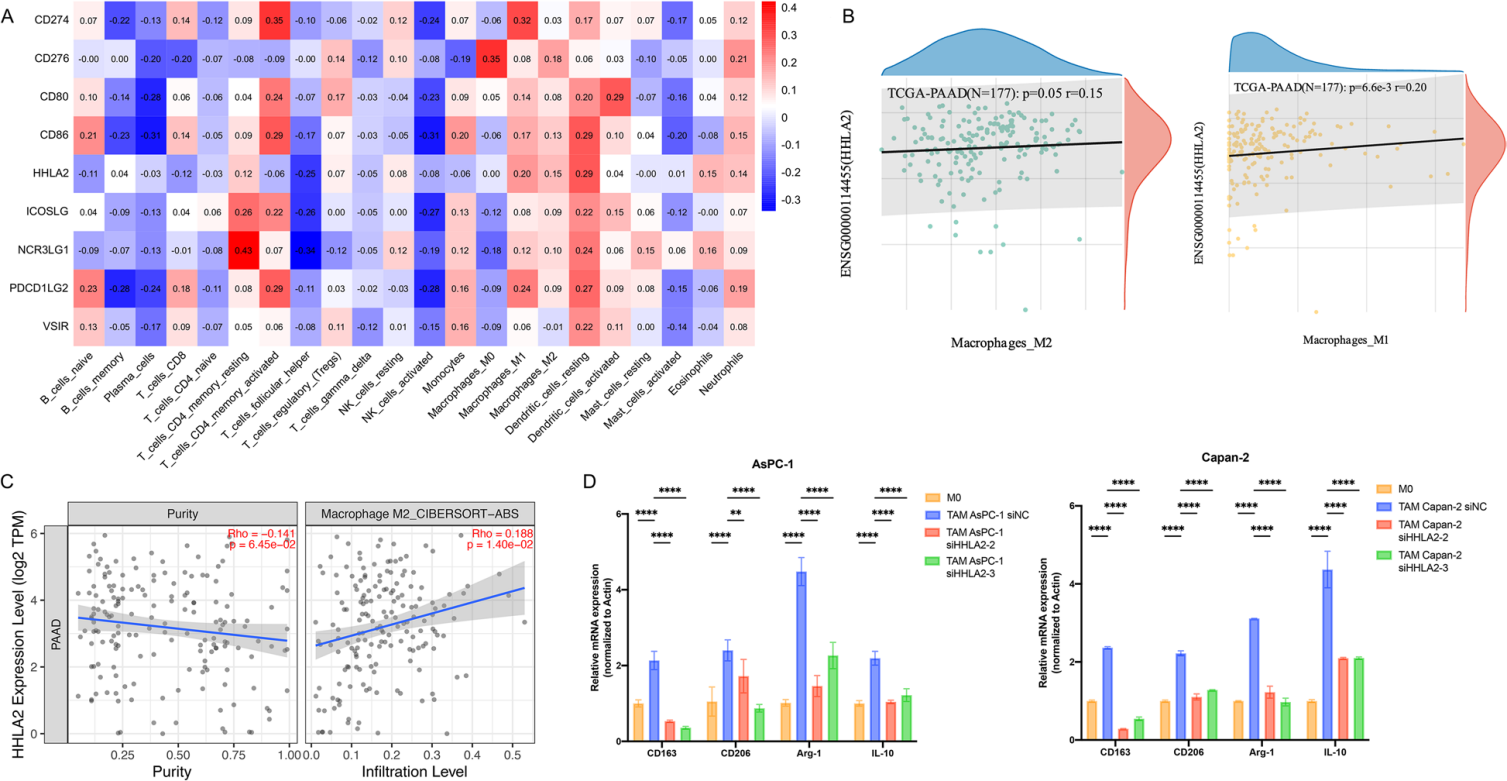
Silencing HHLA2 in AsPC-1 and Capan-2 inhibited M2 polarization of TAMs. **A-C** TIMER2.0 and TCGA database suggested that M2 macrophage infiltration in pancreatic tissue was positively correlated with HHLA2 expression; **D** M0 macrophages and co-cultured TAMs expressed changes in the expression levels of M2-type macrophage markers. ^*^p<0.05, ^**^p<0.01, ^***^p<0.001, ^****^p<0.0001

Finally, effects of HHLA2 on polarization of TAM were examined. We performed the immune infiltration score for the TCGA database through CIBERSORT algorithm, and we calculated the spearman correlation coefficient of the B7 family genes with the immune invasion score to identify significantly correlated immune invasion scores (Fig. 8A). We ultimately observed a significant correlation between B7 family gene expression and immune invasion in TCGA-PAAD. TCGA and TIMER2.0 database showed that M2 macrophage infiltration was positively correlated with HHLA2 expression in PC (Fig. 8B&C). To further explore whether silencing of HHLA2 in PC cell lines was involved in the polarization of TAMs, siHHLA2 or NC was transfected into AsPC-1 and Capan-2 cells. The M0 macrophages were then incubated with conditional medium from NC and siHHLA2 cells for 48 h, and the expressions of M2 markers, CD163, CD206, Arg1 and IL-10 were detected by RT-qPCR. We found the expression levels of CD163, CD206, Arg-1 and IL-10 were significantly up-regulated in the cells of siHHLA2 NC transfected AsPC-1 and Capan-2 compared with M0 group; meanwhile, the expressions of the above markers were partially inhibited by the transfection of siHHLA2-1 and siHHLA2-2 in AsPC-1 and Capan-2 cells (Fig. 8D).

## Conclusion

During the past decades, investigations on immune checkpoint therapy significantly changed the field of cancer treatment. B7 family is one of the most important co-signaling molecules, which is widely expressed in tumor cells and is closely related to tumor progression [23]. B7 family proteins positively or negatively regulate T cell function and are essential for maintaining immune regulation and self-tolerance. The B7 family is the most promising target for immunotherapy, either alone or in combination with other therapies. As a newly discovered member of the B7 family, HHLA2 is absent in most normal tissues, but is over-expressed in some malignant tumors [24]. In this study, we focused on the biological roles of HHLA2 in PC and the immunomodulatory effects of HHLA2 on macrophage polarization. We discovered that HHLA2 is over-expressed in PC and can inhibit the proliferation, migration and invasion of PC cells, and also block the M2 polarization of TAM through regulating EGFR/MAPK/ERK pathway.

HHLA2 overexpression has been reported to be associated with an increased risk of carcinogenesis. For example, results of a previous study suggested that HHLA2 is up-regulated in gallbladder cancer, and increased expression of HHLA2 may indicate poor prognosis [25]; moreover, the destiny of CD8^+^ TILs was also significantly reduced with increased expression of HHLA2 [26]. In the case of PC, the high expression level of HHLA2 not only reveals the occurrence, development, metastasis, prognosis and immune escape mechanism, but may also suggest that HHLA2 can be used as a potential target for immunotherapy of PC patients [27, 28].

In this study, the expression of B7 family member proteins in pancreatic cancer tissues and adjacent normal tissues were investigated by bioinformatics analysis using TCGA, GTEx and TIMER databases. Meanwhile, the clinical significance of HHLA2 in PC was further investigated; furthermore, the effects of HHLA2 on PC cell behaviors were also examined. We found that HHLA2 in over-expressed in PC tissues and cells, moreover, results of cell studies confirmed the oncogenic roles of HHLA2. Taken together, these results suggested that HHLA2 may regulate the growth and metathesis of PC cells, however, the underlying mechanism remains to be investigated.

Epithelial-mesenchymal transition (EMT) refers to the changes that occur in cells under certain physiological and pathological conditions. EMT plays an important role in cell embryonic development, organ differentiation, wound healing, and tumor metastasis [29]. Invasion and metastasis of pancreatic cancer are closely related to EMT [30–33]. Activation of the EGFR/MAPK/mTOR/AKT/ERK1/2 signaling pathway has been reported to promote cancer cell growth, survival, and metabolism [34]. Aberrant activation of the EGFR/MAPK/mTOR/AKT/ERK1/2 signaling pathway occurs in a variety of human cancer cells [35]. EGFR is a tyrosine kinase-type receptor that is commonly activated or overexpressed in a variety of cancers, and the MAPK/ERK signaling pathway mediates the biological effects of EGFR activation [36, 37]. The AKT-mTOR signaling cascade is involved in several physiological and pathological processes, and mTOR is one of the downstream kinases of the AKT signaling pathway. In addition, activation of the EGFR/MAPK/mTOR/AKT/ERK1/2 signaling pathway in various cancers, such as glioblastoma, endometrial cancer, and prostate cancer can promote cancer cell invasion, metastasis, and drug resistance. In our study, we found that silencing of HHLA2 can inhibit the EMT process of pancreatic cancer cells, and the mechanism may be related to the regulation of EGFR/MAPK /mTOR/AKT/ERK1/2 signaling pathway.

Previously, most of the ideas for the treatment of cancer were focused on affecting the behaviors of the tumor cells. In recent years, the roles of tumor microenvironment has attracted more and more attentions. Tumor-associated macrophages (TAMs) refer to a type of macrophages that gather to the periphery of tumor tissues and undergo specific differentiation in the tumor microenvironment under the action of related chemokines [38]. Primitive macrophages (M0 type) can be polarized into M1 or M2 macrophages under different conditions [39]. TAMs are mostly M2 type, which promote tumor growth, invasion and metastasis by secreting a variety of active substances. At present, there are mainly two approaches for blocking tumor-associated macrophages: to inhibit the accumulation of monocytes/tumor-associated macrophages at the tumor site, or to affect the polarization of tumor-associated macrophages to the anti-tumor subtype. In our study, the tumor-infiltrating immune cell model showed that the level of HHLA2 was positively correlated with TAMs in PC, suggesting that HHLA2 was associated with TAMs. Also, we found that after silencing HHLA2 in AsPC-1 and Capan-2 cells, the expression of M2 macrophage markers were significantly decreased. These results indicated that the M2-type polarization of TAMs can be inhibited after silencing HHLA2. The current study also has limitations. HHLA2 is a member of the B7 family that expressed only in humans but not in mice, therefore, the current study only included human samples and human cell lines. In future study, the in vivo analysis may also be conducted by using humanized-mice models.

In conclusion, we found that the expression of HHLA2 is up-regulated in PC tissues, and silencing of HHLA2 can inhibit the proliferation, migration and invasion of PC cells. Meanwhile, silencing HHLA2 can block the EMT process and inhibit the invasion of PC cells, and its mechanism may be related to the regulation of mTOR/AKT and EGFR/MAPK/ERK signaling pathway. Finally, silencing of HHLA2 also inhibited the M2 polarization of TAMs. Our data may provide new evidence for targeting HHLA2 as a novel approach for the management of PC.

### Supplementary Information

Supplementary Material 1: Supplemental table.  Relationship between HHLA2 and clinical features of patients with pancreatic cancer(a. 171 pancreatic cancer patients obtained from the TCGA database; b. Self-made pancreatic cancer tissue microarray).

## Data Availability

No datasets were generated or analysed during the current study.
