# Supplementary material for: HHLA2 deficiency inhibits pancreatic cancer progression and THP-1 macrophage M2 polarization via EGFR/MAPK/ERK and mTOR/AKT pathway
Source: World J Surg Oncol. 2024 May 18;22:133. doi: 10.1186/s12957-024-03409-2 (PMC11102221; doi:10.1186/s12957-024-03409-2)
Supplement: Supplementary file 1 — Supplementary Material 1: Supplemental table. Relationship between HHLA2 and clinical features of patients with pancreatic cancer(a. 171 pancreatic cancer patients obtained from the TCGA database; b. Self-made pancreatic cancer tissue microarray). [file 12957_2024_3409_MOESM1_ESM.docx]

**Supplemental Table**: Relationship between HHLA2 and clinical features of patients with pancreatic cancer（A. 171 pancreatic cancer patients obtained from the TCGA database; B. Self-made pancreatic cancer tissue microarray）

A.

| B. |
| --- |
